# Inhaled predatory bacteria-loaded large porous microspheres to eradicate drug-resistant *Pseudomonas aeruginosa* from the lung

**DOI:** 10.1016/j.mtbio.2025.101562

**Published:** 2025-02-08

**Authors:** Yan Liu, Wanmei Wang, Ruiteng Li, Hui Zhang, Wanting Guo, Bochuan Yuan, Lina Du, Yiguang Jin

**Affiliations:** a Beijing Institute of Radiation Medicine, Beijing, 100850, China; b School of Pharmacy, Xuzhou Medical University, Xuzhou, 221004, China

**Keywords:** Predatory bacteria, *Bdellovibrio bacteriovorus*, Large porous microsphere, Pulmonary delivery, Drug-resistant *Pseudomonas aeruginosa*

## Abstract

The pneumonia caused by antimicrobial-resistant Gram-negative bacteria is an intractable clinical problem due to the lack of effective treatments. Inspired by the unique predatory bacterial ability of *Bdellovibrio bacteriovorus*, we here developed an inhalable live bacterial formulations, i.e., *B. bacteriovorus*-loaded poly(lactic-co-glycolic acid) (PLGA) large porous microspheres (BPMs), to eradicate antimicrobial-resistant *Pseudomonas aeruginosa* from the lung. BPMs serve as a "safe house" of *B. bacteriovorus* to avoid being phagocytized by macrophages due to their large size; while the continual release of *B. bacteriovorus* at the infection site is achieved. We proved BPMs had good biosafety, pulmonary inhalation properties, and antimicrobial effects. The infected mice showed reduced inflammation and lung injury and their respiratory function was well recovered. BPMs have great potential as dry powder inhalers for the treatment of bacterial pneumonia. Inhaled BPMs are an effective treatment against drug-resistant bacterial pneumonia and this live medication is expected to be an alternative therapy to antibiotics.

## Highlights

• *Bdellovibrio bacteriovorus*-loaded porous microspheres (BPMs) formed from poly(lactic-co-glycolic acid).
• The ability of BPMs to protect *B. bacteriovorus* from alveolar macrophage uptake.
• Highly effective anti-drug-resistant bacterial pneumonia with one-shot BPMs treatment.

## Graphical abstract

**Figure:**
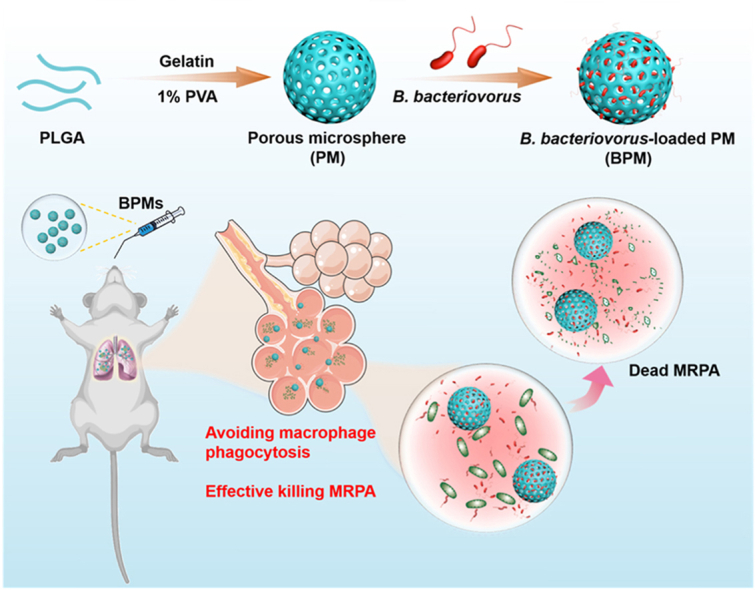

## 1 Introduction

Drug-resistant infections are a severe public health issue with high morbidity and mortality [[1], [2], [3]]. Antibiotics are still the clinically major tool against bacterial infections but the long-term and irregular use of them leads to rising of antibiotic-resistant bacteria [4,5]. The global antimicrobial resistance problem will cost up to $100 trillion by 2050 and the death rate caused by superbugs will exceed that of cancer [6]. The World Health Organization (WHO) identifies four of the most dangerous bacteria, including *Pseudomonas aeruginosa*, due to their widespread nature and high frequency of drug resistance [7]. *P. aeruginosa* is an opportunistic pathogen that can cause a variety of acute infections in hospital settings, such as pneumonia, wound infections, and urinary tract infections [8]. In particular, antimicrobial-resistant *P. aeruginosa* is a difficult-to-treat pathogen in pulmonary infections [9,10], and it is considered as a major threat to pneumonia patients due to its associated morbidity, mortality, and healthcare costs [11,12]. The treatment of antimicrobial-resistant *P. aeruginosa* pneumonia is challenging if only limited antibiotic options are available. Therefore, it is imperative to develop novel therapeutic approaches for treating pneumonia caused by antimicrobial-resistant *P. aeruginosa*.

*Bdellovibrio bacteriovorus* is a small active motile Gram-negative bacterium. It is considered a natural predator due to its ability to prey on a wide range of Gram-negative bacteria such as *Escherichia coli*, *P. aeruginosa*, *Vibrio vulnificus* [[13], [14], [15]]. Since the mechanism by which *B. bacteriovorus* targets bacteria is independent of the action of antibiotics, *B. bacteriovorus* therapy is effective against antimicrobial-resistant bacteria [[16], [17], [18]]. In brief, the bactericidal mechanism of *B. bacteriovorus* is that after encountering the host bacteria, *B. bacteriovorus* enters the periplasmic space of the host bacteria, where it then grows and divides to eventually kill the host bacteria [19,20]. More importantly, *B. bacteriovorus* cannot invade mammalian cells and has high safety [[21], [22], [23]]. Some studies have shown that *B. bacteriovorus* can be readily isolated from the feces and gastrointestinal tract of mammals [[24], [25], [26]]. Therefore, *B. bacteriovorus* is a promising agent against antimicrobial-resistant bacteria, such as meropenem-resistant *P. aeruginosa* (MRPA). Several studies have shown the potential of *B. bacteriovorus* therapy to reduce pulmonary bacterial infections [14,27,28]. Despite the above advantages, *B. bacteriovorus* is mainly used directly as suspensions, which makes it difficult to achieve satisfactory antibacterial effects due to the complexity and variability of the biological environment. Therefore, in order to improve the antibacterial efficacy of *B. bacteriovorus*, it is necessary to develop a suitable formulation strategy for *B. bacteriovorus*. There have been some studies on *B. bacteriovorus* formulations. For example, cryomicroneedles and in situ forming hydrogels for the ocular delivery of *B. bacteriovorus* were successfully prepared with good therapeutic effects on ocular infections [29,30]. Besides, a *B. bacteriovorus*-loaded polyvinyl alcohol/alginate hydrogel was constructed for the topical treatment of the seawater immersion wounds [13]. Furthermore, a material-assisted microorganism strategy that combined high-speed collision-capable predator *Bdellovibrio* with mechanically responsive ZnO nanorods was proposed to treat periodontitis [31]. The formulation strategies described above are designed to deliver *B. bacteriovorus* to the site of infection, maintain its activity, and exert antibacterial effects.

Pulmonary delivery of drugs is a non-invasive method for the treatment of lung diseases, involving lung infections [32,33], pulmonary fibrosis [34], asthma [35], and acute lung injury [36,37]. This route offers several advantages over other administration routes including rapid onset of the action, lung-targeted delivery, little side effects, and high bioavailability [38]. It provides an ideal treatment for pulmonary infections compared to the oral and intravenous routes. Recently, large porous microspheres (PMs) characterized by large geometric diameters, but low density due to high porosity, and small aerodynamic diameters represent an outstanding approach for lung delivery and bypass alveolar macrophage clearance [39,40]. Poly(lactic-co-glycolic acid) (PLGA) has been widely used for the preparation of PMs [[41], [42], [43]]. Such PMs are used for the delivery of various chemical therapeutics [42,44,45], but their use for delivery of living bacteria has not been reported.

Here, we developed porous PLGA microspheres to load *B. bacteriovorus* for pulmonary delivery against MRPA in the lung. The microspheres can be applied for oral inhalation to directly distribute into the deep site of the lung with some advantages, of which are the avoidance of macrophage uptake due to the large size of microspheres, providing a “safe house” for *B. bacteriovorus* to avoid the immune elimination, and becoming a base for the controlled release of *B. bacteriovorus*. Porous PLGA microspheres were prepared with an emulsion-solvent evaporation method using gelatin as the porogenic agent and octadecylamine as the charge regulator. *B. bacteriovorus* porous microspheres (BPMs) were obtained by adsorption of *B. bacteriovorus* based on the geometric and charge effects of PMs. The morphology, pulmonary delivery efficiency, release profile, safety, and *in vitro* antibacterial activity of BPMs were investigated. The therapeutic effect of BPMs on the pneumonia infected with MRPA was evaluated in details. This study provides a solution to the lack of suitable carriers for the deep lung delivery of "live biodrugs" (e.g., *B. bacteriovorus*) and promotes their clinical application.

## 2 Materials and methods

### 2.1 Materials

PLGA (lactide/glycolide, 75:25, mol/mol, MW 20 kDa) was produced by Jinan Daigang Biomaterial Co., Ltd. (Shandong, China). Polyvinyl alcohol (PVA, MW 72,600−81,400 Da, 86 %–90 % hydrolyzed) was purchased from Shanghai Chenqi Chemical Tech. Co., Ltd. (Shanghai, China). Gelatin was purchased from Sigma-Aldrich (St. Louis, Missouri, USA). Octadecylamine was obtained from Beijing InnoChem Tech. Co., Ltd. (Beijing, China). Cy7 was purchased from Xi'an Ruixi Biotechnology Co., Ltd. (Xi 'an, China). Nile Red was purchased from J&K Scientific Ltd. (Beijing, China). A Luria‒Bertani culture medium was purchased from Beijing Solarbio Science & Technology Co., Ltd. (Beijing, China). DMEM culture media, fetal bovine serum (FBS) and trypsin-ethylene diamine tetraacetic acid (EDTA) were purchased from Gibco Life Technologies (Carlsbad, USA). Bronchial epithelial cell media (BEpiCM) were purchased from ScienCell Research Laboratories, Inc. (Carlsbad, CA). Cell Counting Kit-8 (CCK-8) was purchased from Gen-view Scientific Inc. (Tallahassee, USA). *B. bacteriovorus* (ATCC 15356) were purchased from Biobw Biotechnology Co., Ltd. (Beijing, China). MRPA was obtained from clinical isolates. *P. aeruginosa* (ATCC 9027) was provided by Guangdong Microbial Culture Collection Center (Guangzhou, China). Enhanced green fluorescence protein-*E. coli* (eGFP-*E. coli*) was purchased from OBiO Technology Co., Ltd. (Shanghai, China). Meropenem was purchased from Shenzhen Haibin Pharmaceutical Co., Ltd. (Shenzhen, China). SYBR quantitative polymerase chain reaction (qPCR) Master Mix and Taq Master Mix were purchased from Vazyme Biotech Co., Ltd. (Nanjing, China). Other reagents were of analytic grade.

### 2.2 Cells and animals

Normal human bronchial epithelial cells (BEAS-2B cells) were provided by Beijing Institute of Radiation Medicine (Beijing, China), which were grown in the BEpiCM media and incubated at 37 °C in the humidified 5 % CO_2_ environment. Mouse monocyte/macrophage cells (RAW264.7) were purchased from the Cell Bank of the Chinese Academy of Sciences (Shanghai, China), and cultured in the DMEM supplemented with 10 % FBS at 37 °C in a humidified 5 % CO_2_ atmosphere.

Male BALB/c mice (18–20 g) and male Sprague-Dawley (SD) rats (180–200 g) were purchased from the SPF Biotechnology Co., Ltd. (Beijing, China). Animals were housed under the constant conditions of humidity (50 ± 5 %) and temperature (25 ± 1 °C) with 12‒12 h light‒dark cycles. Food and water were available ad libitum. All experimental procedures were approved by the Animal Care and Use Committee of Beijing Institute of Radiation Medicine and complied with the principles of laboratory animal care and use guidelines (IACUC-DWZX-2020-534).

### 2.3 Preparation of BPMs

PMs were prepared using an emulsion/evaporation method as our previous research with modification. Briefly, PLGA (150 mg) and octadecylamine (9 mg) were dissolved in dichloromethane (DCM, 3 mL), and then mixed with a gelatin aqueous solution (7.5 %, 1 mL). A W/O emulsion was obtained after 800-W probe-type sonication for 90 s in an ice bath. The emulsion was mixed with a PVA solution (1 %, 40 mL) and then homogenized to obtain W/O/W emulsions. DCM was removed after stirring overnight to obtain solid microspheres. The suspensions were transferred to 40 °C deionized water and stirred for 3 h to dissolve gelatin. PMs were collected after centrifugation, washed with water, and lyophilized. A *B. bacteriovorus* (2 × 10^9^ PFU/mL) suspension (3 mL) was added to the PMs (5 mg) to obtain bacteria-loaded PMs based on geometrical and charge adsorption effects. BPMs were prepared after centrifugation, washed, and lyophilized. The Nile red-labeled BPMs, Cy7-labeled BPMs, and Cy7-labeled conventional solid PLGA microspheres without gelatin were prepared according to the above procedure. Blank PMs were also prepared without *B. bacteriovorus*.

### 2.4 Characterization of *B. bacteriovorus* and BPMs

The morphology of *B. bacteriovorus* was observed under a transmission electron microscope (TEM, H-7650, 80 kV, Hitachi, Tokyo, Japan). One drop (5 μL) of the suspension containing *B. bacteriovorus* and MRPA was placed on a microscopic copper grid and the excess liquid was removed using filter paper. The sample was then negatively stained with a 5 % phosphotungstic acid solution (pH 7.0) for 3 min and air-dried followed by observation under the TEM.

The morphology of PMs and BPMs was also investigated under scanning electron microscopes (SEM, CUBE II, 20 kV, EmCrafts, Gyeonggi-do, Korea). Briefly, the microspheres were adhered to conductive glue and observed under the SEM after gold spraying.

Volume diameter was defined as geometric median diameter (D_50_) and measured using a particle size analyzer (BT2001, Bettersize Instruments Ltd., Dandong, China) based on the laser light diffraction method. The microspheres were dispersed in water to prepare suspensions for measurement of zeta potentials with Malvern Zetasizer Nano ZS (Malvern, UK) at 25 °C.

To evaluate the bacteria loading efficiency of PMs, we used eGFP-*E. coli* that was incubated with PMs, then filtered through 3.0-μm filters. The resulting microspheres were resuspended and observed using a Nikon Eclipse Ti fluorescent microscope (Tokyo, Japan). PMs were used as the control.

The simulated lung deposition of BPMs was explored with Next Generation Impactor (NGI, TPK 2000R, Copley, Nottingham, UK) with an inspiratory flow rate of 60 L/min. Approximately 10 mg of Nile red-labeled BPM powders were filled in hydroxypropyl methylcellulose hard capsules (Capsugel®, Type 3, Suzhou Capsule Ltd., Suzhou, China) and placed into a DPI device (Type 006, Shanghai Huarui Aerosol Co., Ltd., Shanghai, China) for the deposition test. Deposition was repeated with 10 capsules. The deposited powders were collected from each stage and separately dissolved in methanol for measurement of Nile red with a fluorescence microplate reader (Thermo Scientific, New York, USA). Fine particle fraction (FPF), and mass median aerodynamic diameter (MMAD) were calculated using the software (Copley Inhaler Testing Date Analysis Software, Version 3.10 Wibu USP 32/Ph. Eur.6.0, Copley, Nottingham, UK).

### 2.5 Cytotoxicity test

Cytotoxicity of formulations was further evaluated with the CCK-8 assay method. BEAS-2B cells (5 × 10^3^ cells/well) were seeded in 96-well plates and incubated overnight for adhesion. The *B. bacteriovorus*, PMs, and BPM suspensions were separately prepared with cell culture media and added to the wells followed by incubation for 24 h. The culture media were replaced with 100 μL of 10 % CCK-8 reagents. After incubation for 2 h at 37 °C, optical density (OD) values were measured at 450 nm using a Biotek ELx800 plate reader (Biotek Instruments, Winooski, VT, USA). Cell viabilities were calculated according to the following formula.

$$\text{Cell}\;\text{viability}\;\left(\%\right)=\left({\text{OD}}_{\text{sample}}\;–\;{\text{OD}}_{\text{blank}}\right)\;/\;\left({\text{OD}}_{\text{control}}\;–\;{\text{OD}}_{\text{blank}}\right)\times 100$$

### 2.6 Hemolysis assay

Hemolysis assays of formulations were conducted with mouse's red blood cells (RBCs). Briefly, 2 % RBC suspensions were mixed with *B. bacteriovorus*, PMs, BPMs, saline (negative control), and 1 % Triton X-100 solutions (positive control) at 37 °C for 2 h, respectively. After the suspensions were centrifuged at 2000 rpm and 4 °C for 10 min (H2-16 KR, Hunan Kecheng Instrument and Equipment Co., Ltd., Changsha, China), ODs of supernatants were measured with a UV–Vis spectrophotometer (TU-1901, Beijing Purkinje General Instrument Co., Ltd., Beijing, China) at 540 nm. Hemolysis rates were calculated according to the following formula.

$$\text{Hemolysis}\;\text{rate}\;\left(\%\right)=\left({\text{OD}}_{\text{sample}}\;–\;{\text{OD}}_{\text{negative}}\right)\;/\;\left({\text{OD}}_{\text{positive}}\;–\;{\text{OD}}_{\text{negative}}\right)\times 100$$

### 2.7 *In vivo* safety evaluation

Mice were randomly divided into 6 groups (3 mice/group). Except for one group without treatment as the control group, the other five groups involved intratracheal (i.t.) administration of 20 μL of *B. bacteriovorus* suspensions (2 × 10^9^ PFU/mL), PM suspensions (10 mg/mL, 30 mg/mL), and BPM suspensions (10 mg/mL, 30 mg/mL, wherein the concentrations of *B. bacteriovorus* separately are 1 × 10^9^ PFU/mL, 3 × 10^9^ PFU/mL), respectively. All the mice in each group were sacrificed at 24 h post administration. The lung tissues were excised, and histopathological sections and hematoxylin and eosin (H&E) staining were performed.

### 2.8 *In vitro* release of *B. bacteriovorus* from BPMs

Two milligram of BPMs was added to 3 mL of phosphate buffered solutions (PBS) followed by gentle shaking at 100 rpm and at 37 °C. An aliquot (200 μL) of release media was withdrawn and supplemented with equal volumes of PBS at the predetermined time points (0.25, 0.5, 1, 2, 4, 8, 12, 24, and 48 h), and the released *B. bacteriovorus* was determined with the qPCR method. Additionally, the surface morphologies of the microspheres at different time points were investigated with SEM.

### 2.9 Antibacterial experiment

A volume of 200 μL of HEPES buffer (control), *B. bacteriovorus* suspensions (1 × 10^8^ PFU/mL), PM suspensions (1 mg/mL), BPM suspensions (1 mg/mL, wherein the concentration of *B. bacteriovorus* is 1 × 10^8^ PFU/mL), and meropenem solutions (16 μg/mL) were added to 2 mL of MRPA HEPES suspensions (1 × 10^9^ CFU/mL), and then co-cultured in a shaker at 180 rpm and 37 °C. After 24 h, the appearance of the bacterial suspensions was observed and the OD values at 630 nm were measured with the microplate reader (Spark, Tecan Group Ltd., Switzerland). Moreover, a series of fresh BPMs were preserved at 4 °C for 3, 5, and 7 days, respectively, and their antibacterial abilities were investigated as above.

### 2.10 Uptake by macrophages

Macrophage uptake of BPMs was evaluated by co-incubation of the Nile red-labeled BPMs and mouse macrophages. RAW264.7 cells were incubated in 6-well plates at 37 °C for 12 h. BPMs (1 mg) were suspended in the DMEM culture media and added into each well followed by incubation. The plates were observed under a fluorescence microscope (Eclipse Ti, Nikon, Tokyo, Japan) at predetermined time points. As the mentioned above process, BPMs were incubated with the media without cells and centrifuged at 12,000 rpm for 10 min. The precipitated particles were lyophilized to obtain the powders that were observed under the SEM as above.

### 2.11 *In vivo* lung deposition study

In the *in vivo* deposition experiments, Cy7-labeled BPMs were quickly administered to the rat lung using an insufflator (DP-4, Penn-Century Inc., PA, USA) through the trachea without anesthesia. To confirm the lung deposition of microspheres, the whole lung was observed with an *in vivo* imaging system (IVIS, Lumina II, Caliper Life Sciences, Waltham, USA). The lung tissue sections were investigated using the fluorescence microscope. Cy7-labeled non-porous PLGA solid microspheres were used as the control to compare the lung deposition efficiency with BPMs.

### 2.12 Establishment of mouse pneumonic models and therapy

Mouse bacterial pneumonic models were established as referred to our previous work. Briefly, mice were anesthetized and fixed on a mouse operating table (HRH-HAG6, Beijing Huironghe Technology Co., Ltd., China). A laryngoscope (HRH-HAG5, Beijing Huironghe Technology Co., Ltd., China) was used to visualize tracheal opening. An aliquot (25 μL) of 1 × 10^6^ CFU/mL MRPA or wild-type *P. aeruginosa* (WTPA) suspensions was sprayed into the lung of mice through the trachea using the MicroSprayer®/Syringe Assembly including an IA-1B MicroSprayer® Aerosolizer and an FMJ-250 High-Pressure Syringe (Penn-Century Inc., PA, USA). Treatment was performed 4 h pre- and 2 h post-modeling.

The mice were divided equally into eight groups and six mice of each, including the healthy, model, WTPA plus meropenem, MRPA plus meropenem, WTPA plus *B. bacteriovorus*, MRPA plus *B. bacteriovorus*, WTPA plus BPMs, and MRPA plus BPMs groups. Except for i.t. administration of saline (25 μL) for each mice in the healthy and model groups, the equal volume of meropenem solutions (0.5 mg/mL), *B. bacteriovorus* suspensions (1 × 10^9^ PFU/mL), and BPM suspensions (10 mg/mL) were separately i.t. administered to the other mice according to the above administration schedule. Physiological and pathological tests began 24 h post the last treatment, involving respiratory function, movement ability, and oxygen saturation (SpO_2_) levels. And then, the lung tissues of the mice were excised after sacrifice, where the upper lobes of the right lungs were immersed in paraformaldehyde buffers (4 %, w/v) for the following histopathological studies, and the middle and lower lobes of the right lungs were used for measurement of pro-inflammatory cytokines. The left lungs were used to measure bacterial loads.

### 2.13 Assessment of physiological functions

An unrestrained whole-body plethysmograph (EMKA Technologies, Paris, France) was used for measurement of mid-expiratory flow rate (EF50) and tidal volume (TV) of the mice. Respiratory indexes were automatically recorded for 5 min. A finger clip pulse oximeter (MD300C, Beijing Chaosi Electronic Technology Co., Ltd., Beijing, China) was clamped at the tail of mice for measurement of SpO_2_. The physical activity of mice was evaluated with a rotating rod fatigue tester (DB093, Beijing Zhishu Duobao Biological Technology Co., Ltd., Beijing, China). Each mouse was trained at 10 rpm for 15 s before the experiment began. The total test time was set to 120 s. The rotation speed of rod (93 mm in diameter) gradually increased from 0 rpm to 20 rpm within 20 s and maintained to the end. The number of dropping times was recorded.

### 2.14 Determination of bacterial loads in the lung

A qPCR method was used to quantitatively determine the content of *P. aeruginosa* in the lung tissues. The primers of *P. aeruginosa* (forward primer: 5′-TTGGGAGGAAGGGCAGTAAG-3′; reverse primer: 5′-CTCTACCGTACTCTAGCTCAG-3′) were designed with SnapGene software. The left lungs of mice were processed to extract tissue DNA according to the instruction of the kit (E.Z.N.A.®, Omega Bio-Tek, Inc., USA). Each qPCR reaction contained 10 μL of qPCR Master Mix (2 × Taq Pro Universal SYBR, Vazyme), 1 μL of template, and 0.4 μL of each primer (10 μmol/L) to 20 μL. The qPCR cycle conditions were as follows: 3 min at 95 °C, followed by 40 cycles of 10 s at 95 °C, 30 s at 60 °C, 30 s at 72 °C, 15 s at 95 °C, 60 s at 60 °C, and 15 s at 95 °C. At the same time, a series of *P. aeruginosa* suspensions were used as templates for qPCR assay. A standard curve for C_t_ (threshold cycle) values and *P. aeruginosa* copy numbers was established, which was used to calculate the *P. aeruginosa* loads in lung tissues combined with the measured C_t_ values of samples.

### 2.15 Determination of pro-inflammatory cytokines in the lung

The middle and lower lobes of the right lungs of mice were homogenized with ice-cold 0.9 % sterile saline to prepare 10 % (w/v) homogenates. The obtained homogenates were centrifuged for 10 min at 5000×*g* and 4 °C (Kecheng Instrument and Equipment Co., Ltd., China). The supernatants were collected and the concentrations of tumor necrosis factor-α (TNF-α) and interleukin-6 (IL-6) were determined with ELISA kits (Shanghai Enzyme-linked Biotechnology Co., Ltd., China) according to the manufacturer protocols.

### 2.16 Histopathology and immunohistochemistry

The upper lobes of the right lungs of the mice were fixed with 4 % paraformaldehyde and then embedded in paraffin. Embedded lungs were cut into standard sections followed by H&E staining. Some sections were used for immunohistochemical labeling of nuclear factor-κB (NF-κB) p65, and inflammasome NLRP1. Histological sections of the lung tissues were finally examined under a microscope (BDS200-FL, Chongqing Optec Instrument Co., Ltd., Chongqing, China).

### 2.17 Apoptosis assay of lung tissues

The above paraffin-embedded upper lobes of the right lungs were used for apoptosis assay. The terminal deoxynucleotidyl transferase biotin-dUTP nick end label (TUNEL, Beijing Solarbio Science & Technology Co., Ltd., Beijing, China) staining was conducted followed by incubation of tissue sections for 60 min at 37 °C. The sections were washed with PBS (pH 7.4), and then incubated with DAPI (4,6-diamidino-2-phenylindole) for 10 min at room temperature for detection of nucleoli. Images of TUNEL and DAPI fluorescence were recorded using a fluorescent microscope. TUNEL staining uses fluorescein as a marker for in situ labelling of 3′ ends exposed by DNA breaks in the cell nucleus, which visually reflects the apoptosis through fluorescence intensity [46].

### 2.18 Statistical analysis

All data expressing as mean ± standard deviation (SD) of at least three replicates were analyzed statistically using SPSS 16.0 software (SPSS, Chicago, IL, USA). One-way analysis of variance (ANOVA) with the LSD test was carried out to compare between multiple groups. Statistically significant differences were represented with ∗*p* < 0.05, ∗∗*p* < 0.01, and ∗∗∗*p* < 0.001.

## 3 Results and discussion

### 3.1 Characteristics of *B. bacteriovorus* and BPMs

**Fig. 1:**
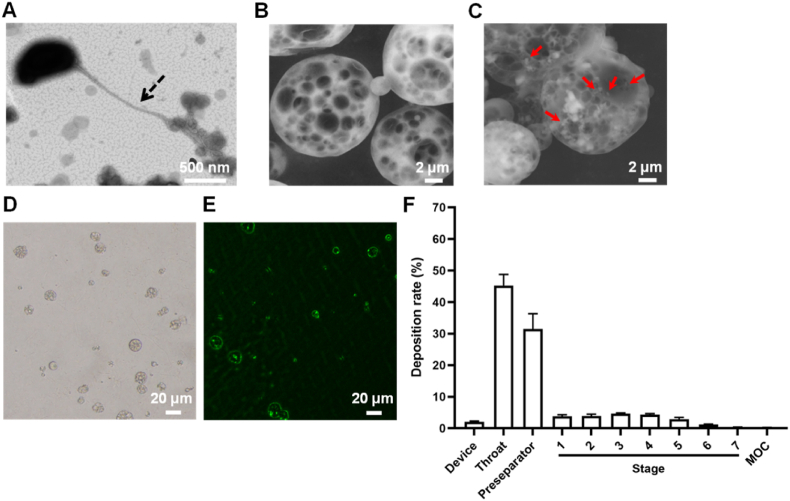
Characteristics of *B. bacteriovorus* and microspheres. TEM image of *B. bacteriovorus* (A). Dotted arrows indicate the flagella of *B. bacteriovorus*. SEM images of PMs (B) and BPMs (C). The red arrows indicate *B. bacteriovorus*. Bright field microscopic image of PMs (D). Fluorescence microscopic image of microspheres after co-incubation of eGFP-*E. coli* and PMs (E). (F) Simulated lung deposition of Nile red-labeled BPMs in the different compartments of NGI (*n* = 3).

*B. bacteriovorus* has a tiny, arc-shaped form, with a size of about 0.4 μm × 0.8 μm, and a long flagellum (Fig. 1A), which is conducive to its rapid swimming and "finding" prey. Although the size of *B. bacteriovorus* was about a quarter of the size of its prey, it did not affect *B. bacteriovorus* feeding on its prey [47,48]. Invasion begins by the production of a small pore in the outer membrane of the prey, through which *B. bacteriovorus* squeezes. Immediately after entry into the prey cell, the pore in the prey's outer membrane is promptly sealed, and the prey cell starts a morphological transition leading to the characteristic round shape of the bdelloplast. Once the entry is completed *B. bacteriovorus* starts to consume all the available nutrients within the bdelloplast in order to replicate itself [49]. We observed that the PMs were approximately 10 μm in size and had a large number of pores (Fig. 1B), and *B. bacteriovorus* was loaded in BPMs (Fig. 1C). The pore size of BPMs was mostly in the range of 2–6 μm, and the large porous structure of the microspheres facilitated the loading, survival and swimming of *B. bacteriovorus*. The average geometric size and zeta potential of the PMs were 9.10 ± 0.14 μm and 11.08 ± 0.69 mV, respectively; while the average geometric size and zeta potential of the BPMs were 10.17 ± 0.21 μm and −3.68 ± 0.87 mV, respectively (Fig. S1). Octadecylamine was added during the preparation of the PMs to make the microspheres positively charged. When co-incubated with *B. bacteriovorus*, the geometric effect and charge adsorption effect of the PMs [50], caused *B. bacteriovorus* to enter the microspheres and adsorb on them, resulting in a change in the zeta potential of the microspheres from positive to negative, and a slight increase in particle size.

The green fluorescence of eGFP-*E. coli* can be observed under the GFP channel of the fluorescence microscope and is often used as a model strain. The PMs with a high number of pores were observed under the bright field of the microscope (Fig. 1D). Obvious green fluorescence accumulated in the PMs that were co-incubated with eGFP-*E. coli* (Fig. 1E), indicating that the PMs were adsorbed or loaded with eGFP-*E. coli*. This was related to the porous geometric effect and positive electrical properties of PMs.

The lung deposition or pulmonary delivery efficiency of particles is important to their biological effect, which is determined by their aerodynamic diameters. The complex and specific physiology of the lung makes it necessary to meet certain conditions for the pulmonary delivery of drug formulations. Particles with an aerodynamic size of 1–5 μm are generally considered to be more suitable for pulmonary administration [51,52]. FPF and MMAD are two crucial factors in the assessment of pulmonary deposition of inhaled powders. The FPF of BPMs was 14.74 %, greater than 10 %, which is the standard set by the Chinese Pharmacopoeia 2020 edition, suggesting a high rate of lung deposition of BPMs. Despite their geometric diameter of 10.17 μm, the MMAD of BPMs was only 3.32 μm, which lay within the range of 1–5 μm, indicating that it can be effectively deposited deeply in the lung (Fig. 1F). Therefore, BPMs can be used as powder aerosols for effective pulmonary delivery.

Large PMs are an optimal selection for loading *B. bacteriovorus* because a great number of microscale holes exist in them. PMs should be manufactured with biodegradable and biocompatible biomaterials. PLGA is a friendly biomaterial and its unique final metabolites are dioxide carbon and water. The large geometric size but small aerodynamic size of PMs makes their well distributing in the lung. Another important factor is how to load *B. bacteriovorus* with high efficacy. A simple lipidic amine, octadecamine, was used as the charge regulator to make PMs carrying cations. Based on the electrostatic adsorption, negatively-charged *B. bacteriovorus* could adsorb on the surface of positively charged PMs or enter the inner space of PMs. Whatever any loading mode, *B. bacteriovorus* should be protected from the phagocytosis of macrophages in the lung due to the large size effect of PMs. Adjusting the additional amount of octadecamine can got the optimal loading of *B. bacteriovorus*.

### 3.2 High safety of BPMs

**Fig. 2:**
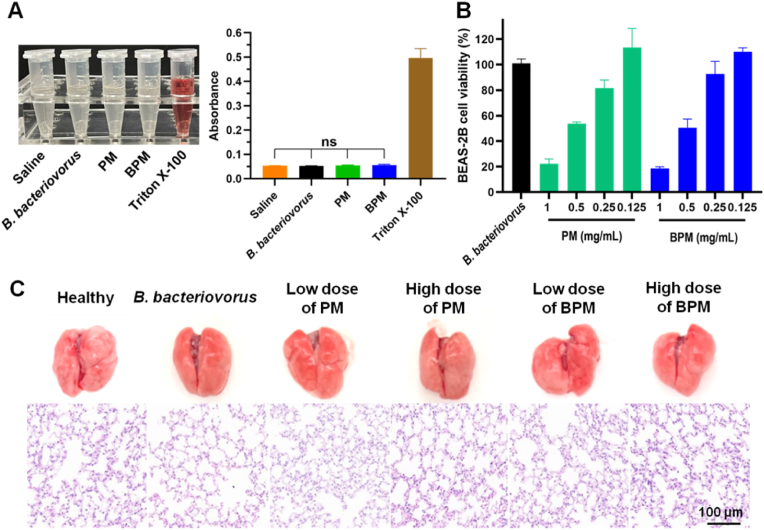
*In vitro* biocompatibility and *in vivo* safety of *B. bacteriovorus* and microspheres. (A) Photographs and absorbances of the supernatants after RBC being mixed with saline (negative control), *B. bacteriovorus*, PMs, BPMs, and Triton X-100 (positive control). (B) BEAS-2B cell viability after co-incubation with *B. bacteriovorus*, different concentrations of PMs, and BPMs. The concentrations indicate the final concentrations of added microspheres in the culture media. (C) Photographs of the lungs and the H&E staining images in different groups. *n* = 3; ns, no significance.

There are many blood vessels in the lung and lung injury is often accompanied by severely damaged alveoli and blood vessels [53,54]. Therefore, it is necessary to investigate the blood compatibility of BPMs. The appearance of the hemolysis assay showed that the *B. bacteriovorus* group, the PM group and the BPM group were similar to the saline group (i.e., the negative control) and their supernatants were clear and transparent without obvious red. In contrast, the supernatant of the Triton X-100 group (i.e., the positive control) was clearly red, indicating the occurrence of hemolysis (Fig. 2A). The *B. bacteriovorus*, PM and BPM groups showed the almost same OD values (ca. 0.05) of the supernatants at 540 nm as the negative control, but the positive control had a high OD value (ca. 0.5) (Fig. 2A). Furthermore, RBCs maintained intact morphology with a double-concave disc shape except for the positive control (Fig. S2). Therefore, BPMs have good blood compatibility without hemolysis.

BEAS-2B cells, a human normal bronchial epithelial cell line, were used to verify the respiratory system toxicity of BPMs. CCK-8 assay showed that *B. bacteriovorus* (1 × 10^8^ PFU/mL) alone, 0.125 mg/mL PM suspensions and 0.125 mg/mL BPM suspensions had almost no effect on the proliferative growth of BEAS-2B cells with high cell viabilities close to 100 %. However, the increase in the doped microspheres caused the decrease in cell viabilities (Fig. 2B). The highly concentrated microspheres could settle at the bottom of the cell culture plates, causing the physical compression of the walled cells and occupying the cell growth space, so that the cell proliferation was inhibited.

In the *in vivo* safety study, *B. bacteriovorus*, PMs, and BPMs were separately i.t. administered to mice. After maintaining for 24 h, the lungs were excised and they were smooth, ruddy, and soft in texture without difference from the healthy lungs (Fig. 2C). H&E stained pathological sections showed that the lung tissues of mice in both the treatment groups and the healthy control group were clearly structured and well-arranged, with intact alveolar cavities, no significant thickening of the alveolar septa, and no inflammatory cell infiltration (Fig. 2C). Therefore, inhaled BPMs had no adverse effects on the lung tissue of mice and had good *in vivo* safety.

*B. bacteriovorus* as a natural potent bacterial predator has been focused on for many years [55,56]. Its major advantage is that Gram-negative bacteria are the unique prey. It does not interfere with mammalian cells except for uptake by our immune cells [57]. The growth of *B. bacteriovorus* is self-limited depending on its preys. *B. bacteriovorus* will disappear if the prey is totally consumed off. So, the safety of *B. bacteriovorus* can be ensured.

### 3.3 Effective predator release and high bactericidal capability of BPMs

**Fig. 3:**
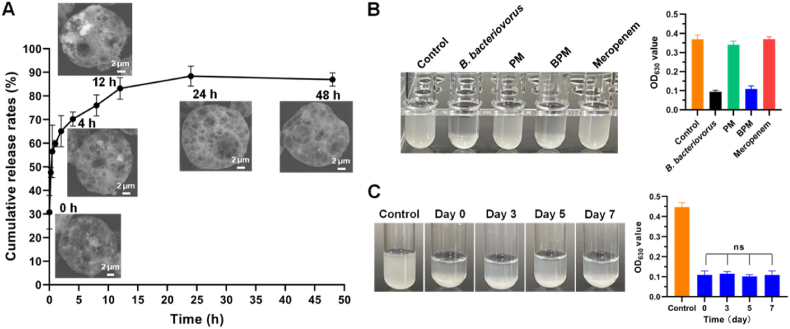
*B. bacteriovorus* release from BPMs and bactericidal capability of BPMs. (A) *In vitro B. bacteriovorus* release profile of BPMs and typical SEM images of BPMs after releasing. (B) Appearance and OD_630_ values of cultivation liquids after co-culture of MRPA with different samples. (C) Appearance and OD_630_ values of the cultivation liquids of BPMs/MRPA after BPMs preservation at 4 °C. *n* = 3; ns, no significance.

After BPMs contacted PBS, *B. bacteriovorus* adsorbing on the surface of BPMs quickly released to the surroundings to achieve 2.67 × 10^7^ PFU/mL (Fig. 3A), which was beneficial to kill the surrounding environmental pathogenic bacteria. As time proceeding, *B. bacteriovorus* locating inside the BPMs gradually released through the pores, reaching a cumulative release rate of about 70 % at 4 h. An 88 % release was achieved and went to an equilibrium at 24 h. The SEM images of BPMs after releasing clearly showed the porous-microspherical structures that maintained for 24 h at least. Finally, PMs would likely be degraded (Fig. 3A). The continual release of *B. bacteriovorus* from BPMs provides high bactericidal capability. In the *in vitro* environment, BPMs had the same high bactericidal capability as *B. bacteriovorus* with clear liquids (low OD_630_ values) after co-incubation with MRPA. But meropenem did not show any bactericidal effect with a cloudy liquid (high OD_630_ values) (Fig. 3B). Moreover, the bactericidal effect of BPMs is kept after long-term preservation. Even after preservation at 4 °C for 7 days, BPMs still maintained strong bactericidal effect, meaning the high activity of *B. bacteriovorus* in BPMs (Fig. 3C).

### 3.4 Avoidance of macrophage phagocytosis and highly effective lung distribution of BPMs

**Fig. 4:**
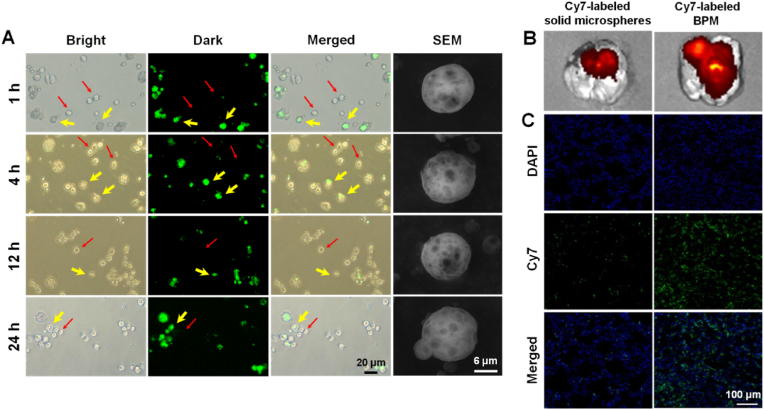
Avoidance of macrophage uptake and highly effective lung distribution of BPMs. (A) Microscopic images and SEM images of the Nile red-labeled BPMs and macrophages RAW264.7 after co-incubation. The red thin arrows indicate the same macrophages shown in the bright, dark, and merged fields, and the yellow thick arrows indicate BPMs. IVIS images (B) and lung tissue section images (C) of the lung distribution of conventional solid microspheres and BPMs after i.t. administration to rats for 4 h.

Macrophages appeared round when unpolarized, as indicated with the red arrows in Fig. 4A. The BPMs labeled with Nile red exhibited green fluorescence in the dark field with the GFP channel of the fluorescence microscope (Fig. 4A), indicated with the yellow arrows. If BPMs were phagocytized by macrophages, the merged images would likely show the overlap of macrophages and BPMs. However, we did not observe overlapped fluorescence within 24 h, indicating no uptake of BPMs by macrophages. Moreover, the SEM images of BPMs showed the complete porous-microspherical structure within 24 h (Fig. 4A). In fact, according to the release profile of BPMs in the above section, *B. bacteriovorus* hardly released after 24 h (Fig. 3A). Therefore, BPMs provide a “safe house” and reservoir for *B. bacteriovorus* against phagocytosis in the lung for a long time, which ensures *B. bacteriovorus* maintaining a certain number in the infected tissue. The macrophage-resistant action of large PMs had been confirmed in our previous research [34,39]. Therefore, BPMs are suitable for pulmonary delivery of *B. bacteriovorus*.

Another advantage of BPMs is their highly effective lung deposition besides their predator reservoir role. A conventional solid microsphere was compared to BPMs after they were labeled with Cy7. Both of them only deposited in the lungs after i.t. administration (Fig. S3). However, the detailed deposition sites of them were very different, wherein BPMs widely distributed in the whole lung but the solid microspheres only distributed in the upper trachea and bronchi (Fig. 4B). The unique porous structure of BPMs contributes the wide distribution due to their small aerodynamic diameters, suitable for pulmonary delivery via oral inhalation. The lung tissue section images further showed the wide distribution of BPMs versus the very little distribution of solid microspheres (Fig. 4C).

It's worth noting that the loading and delivery of *B. bacteriovorus* is a huge challenge because *B. bacteriovorus* would likely be removed by immune cells, like other bacteria. In this study, we designed a novel delivery vehicle to load and transport *B. bacteriovorus* without affecting its release but avoiding the uptake of pulmonary macrophages. More importantly, the biodegradable vehicle, i.e., PMs, can be widely distributed in the lung after inhalation, which provides a very good opportunity to release *B. bacteriovorus* everywhere in the infected tissues. So, we use one-shot administration of BPMs to treat MRPA pneumonia.

### 3.5 Recovery of respiratory functions due to pulmonary delivery of BPMs

**Fig. 5:**
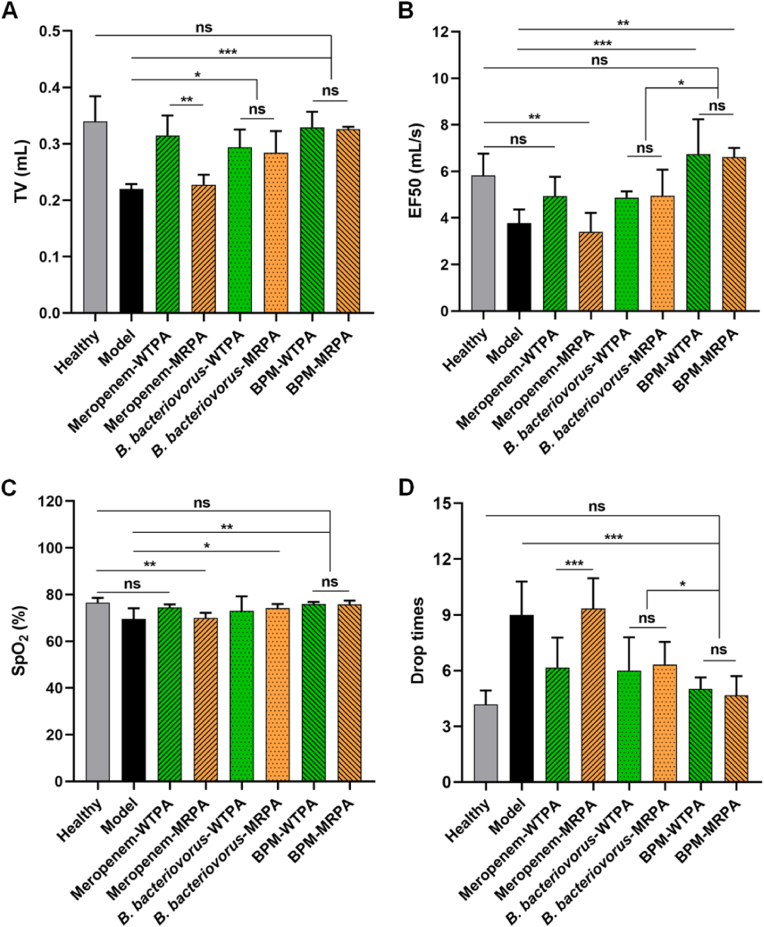
Pulmonary respiratory functions of the mice after different treatments. Pulmonary ventilation function indexes TV (A) and EF50 (B), and pulmonary gas transfer function indexes SpO_2_ (C) and drop times (D) of the mice in different groups. Meropenem-WTPA, *B. bacteriovorus*-WTPA, and BPM-WTPA represent the mice with WTPA pneumonia treated with meropenem solutions, *B. bacteriovorus* suspensions, and BPM suspensions, respectively. Meropenem-MRPA, *B. bacteriovorus*-MRPA, and BPM-MRPA represent the mice with MRPA pneumonia treated with meropenem solutions, *B. bacteriovorus* suspensions, and BPM suspensions, respectively. *n* = 3, ∗*p* < 0.05, ∗∗*p* < 0.01, ∗∗∗*p* < 0.001; ns, no significance.

Pneumonic animals usually own limited respiratory function due to the serious infection and the damage of pulmonary structure. Recovery of respiratory function means the control or elimination of infections in the lung. TV is an indicator of lung volume and its low value indicates inadequate pulmonary ventilation. EF50 is an index of airway obstruction, which indicates the degree of airway resistance or the severity of ventilation disorder. TV and EF50 are important indicators of the respiratory function of the organism [58]. Compared with the WTPA (i.e., non-resistant *P. aeruginosa*) pneumonia treated with meropenem group (meropenem-WTPA), the TV and EF50 of the mice in the MRPA pneumonia treated with meropenem group (meropenem-MRPA) were significantly decreased (Fig. 5A and B), suggesting that antibiotic-resistant bacteria are difficult to treat and pose a threat to health. There was no difference in TV and EF50 between WTPA pneumonia group and MRPA pneumonia group treated with *B. bacteriovorus* (including BPMs), indicating that *B. bacteriovorus* did not distinguish between wild-type bacteria and antibiotic-resistant bacteria. Compared with the model group, the TV and EF50 of BPM groups (including BPM-WTPA and BPM-MRPA) were significantly different (*p* < 0.01, *p* < 0.001), indicating that the respiratory function of mice was greatly improved. Besides, the EF50 values of the *B. bacteriovorus* groups and the BPM groups had statistical difference (*p* < 0.05). In the BPM groups (including BPM-WTPA and BPM-MRPA), the TV and EF50 levels of mice were not significantly different from those of the normal group, indicating that the respiratory function of pneumonic mice had returned to normal. In conclusion, BPMs could effectively alleviate the impaired pulmonary ventilation caused by bacterial infectious pneumonia, without distinguishing whether the bacteria were resistant or wild-type.

SpO_2_, the concentration of oxygen in the blood, is an important physiological parameter of the respiratory cycle, which can reflect whether the lung tissue of the organism is normal to a certain extent. When the body has gas exchange dysfunction, it can lead to a decrease in SpO_2_. In addition, the rotating-stick experiment was used to examine the exercise capacity of mice, which in turn reflected the oxygen supply capacity of the organism. Mice with fewer drops had better locomotor ability. The healthy mice had an SpO_2_ of about 77 %, while the pneumonic mice had a reduced level of SpO_2_ of about 69 % (Fig. 5C). Besides, the pneumonic mice showed much more drop times from the rotating rod fatigue tester than the healthy mice (Fig. 5D). In both BPM-WTPA group and BPM-MRPA group, the SpO_2_ values were not significantly different from the normal group (Fig. 5C). BPMs could prevent the dropping of the mice with WTPA and MRPA pneumonia from the tester (Fig. 5D). Moreover, the drop times of the *B. bacteriovorus* groups and the BPM groups had statistical difference (*p* < 0.05). In all factors, the mean values of the BPM groups were better than the mean values of the *B. bacteriovorus* groups. In summary, the BPM groups had better anti-pneumonia ability than the *B. bacteriovorus* groups. BPMs could attenuate the mouse lung injury caused by wild-type and antimicrobial-resistant *P. aeruginosa* infections, improve the pulmonary respiratory function, and consequently enhance the physical activity.

### 3.6 High *in vivo* antibacterial effect of BPMs and recovery of infected lung tissues

**Fig. 6:**
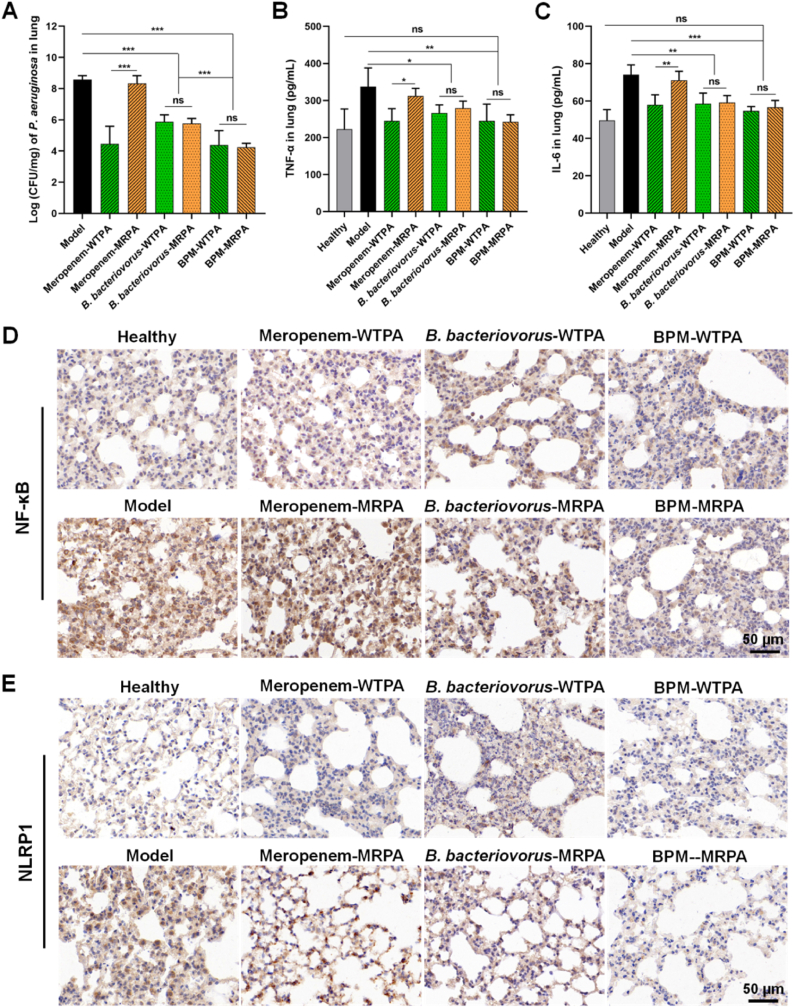
Antibacterial and anti-inflammatory effects of BPMs on the mice with pneumonia. (A) The *P. aeruginosa* numbers in the mouse lungs in the different groups. Expressions of TNF-α (B) and IL-6 (C) in the lung tissues of the mice in the different groups. The images of immunohistochemical staining of NF-κB (D) and NLRP1 (E) in the lung tissues of the mice in the different groups. Meropenem-WTPA, *B. bacteriovorus*-WTPA, and BPM-WTPA represent the mice with WTPA pneumonia treated with meropenem solutions, *B. bacteriovorus* suspensions, and BPM suspensions, respectively. Meropenem-MRPA, *B. bacteriovorus*-MRPA, and BPM-MRPA represent the mice with MRPA pneumonia treated with meropenem solutions, *B. bacteriovorus* suspensions, and BPM suspensions, respectively. *n* = 3, ∗*p* < 0.05, ∗∗*p* < 0.01, ∗∗∗*p* < 0.001; ns, no significance.

The load of *P. aeruginosa* in different groups of samples was obtained by the established real-time qPCR method. Although the bacterial load in the meropenem-WTPA group was much lower than that of the meropenem-MRPA group (*p* < 0.001), there was no significant difference between WTPA pneumonia group and MRPA pneumonia group treated with *B. bacteriovorus* (including BPMs) (Fig. 6A). The *B. bacteriovorus* groups and the model group had great differences in the pathogen amounts in the lungs (*p* < 0.001), indicating that *B. bacteriovorus* showed great antibacterial ability after it entered the lung (Fig. 6A). Moreover, the *B. bacteriovorus* groups and the BPM groups also had great differences (*p* < 0.001), indicating that in BPMs, the loaded *B. bacteriovorus* not only released from the BPMs to kill pathogens, but also maintained a certain amount to prevent the removal by the immune system. Therefore, the inhalation of BPMs was effective in clearing bacteria from the infected lung, and BPMs had good antibacterial abilities against antimicrobial-resistant bacteria *in vivo*.

The inflammatory response often occurs with bacterial infection. Bacterial endotoxins can activate macrophages, and thus produce pro-inflammatory cytokines. Pro-inflammatory cytokines (e.g., TNF-α and IL-6) may up-regulate the inflammatory response, leading to damage to bacterial infected tissues [59]. BPMs highly decreased the expression of TNF-α and IL-6 in the lungs of mice with WTPA pneumonia and MRPA pneumonia (Fig. 6B and C). The values of TNF-α in the BPM groups and the model groups had great differences (*p* < 0.01), but the difference between the *B. bacteriovorus* groups and the model groups was not high (*p* < 0.05). A similar trend was observed for IL-6. In addition, NF-κB is a key transcription factor mediating the release of inflammatory cytokines [60,61]. Inflammasome (e.g., NLRP1) is an intracellular multiprotein complex that can promote the maturation and secretion of inflammatory cytokines, exhibiting pro-inflammatory effects [62]. Immunohistochemical staining for NF-κB protein and NLRP1 was performed in this experiment, and the brown-yellow staining of cells represented positivity. The expressions of NF-κB p65 and NLRP1 were significantly increased in the model group, while the mice with WTPA pneumonia and MRPA pneumonia had the least positive expressions after treated with BPMs (Fig. 6D and E), which was consistent with the expression of pro-inflammatory cytokines TNF-α and IL-6 (Fig. 6B and C).

**Fig. 7:**
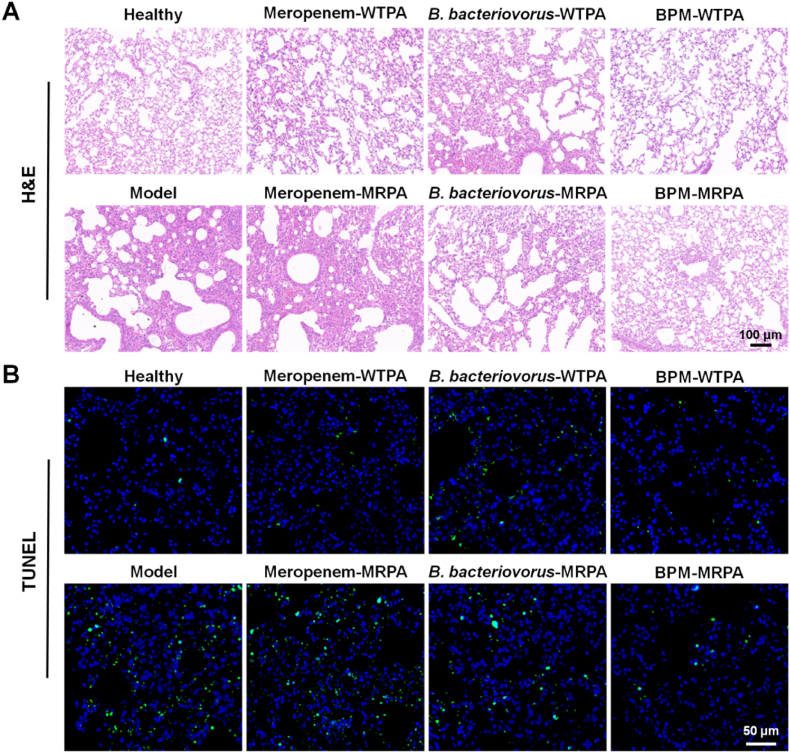
BPMs alleviate injury and apoptosis in lung tissues of mice with pneumonia. (A) H&E staining images of lung tissues in different groups of mice. (B) TUNEL staining images of lung tissues in different groups of mice. Meropenem-WTPA, *B. bacteriovorus*-WTPA, and BPM-WTPA represent mice with WTPA pneumonia treated with meropenem solutions, *B. bacteriovorus* suspensions, and BPM suspensions, respectively. Meropenem-MRPA, *B. bacteriovorus*-MRPA, and BPM-MRPA represent mice with MRPA pneumonia treated with meropenem solutions, *B. bacteriovorus* suspensions, and BPM suspensions, respectively.

Bacterial infections of the lungs can damage lung tissue if not treated in time [63]. Lung appearance and pathological changes are intuitive indicators of lung lesions [64]. The lung tissues of the model group showed obvious hemorrhages and alveolar collapse compared to the healthy lung tissues owning the ruddy and smooth appearance and the clear normal lung alveolar septum (Fig. S4 and Fig. 7A). Meropenem was only effective against WTPA pneumonia, while BPMs had good prevention and treatment effects on both WTPA and MRPA pneumonia. Besides, the therapeutic effect of *B. bacteriovorus* suspensions was not as good as BPMs, probably because of the protection and release of BPMs on *B. bacteriovorus*. Furthermore, the TUNEL staining experiment was carried out. The green fluorescence intensity of meropenem-WTPA group was weak, while that of meropenem-MRPA group was strong, indicating that serious apoptosis occurred after MRPA infection (Fig. 7B). The green fluorescence in the BPM-WTPA and BPM-MRPA groups was little, suggesting that BPMs could reduce the apoptosis of lung tissue cells caused by WTPA and MRPA infections.

In short, pulmonary administration of BPMs could significantly reduce the bacterial loads in lung tissues of mice infected with WTPA and MRPA pneumonia, down-regulate the expression and release of pro-inflammatory cytokines, and alleviate the inflammatory response and lung tissue damage.

## 4 Conclusion

Pneumonia caused by Gram-negative bacteria has become a common clinical disease with a high incidence, seriously affecting public health. Besides novel antibiotics, human can learn from the nature to resist the continual arising of antimicrobial-resistant bacteria. As a living antibiotic, *B. bacteriovorus* could effectively kill most Gram-negative bacteria and is not affected by drug resistance. However, direct pulmonary administration of *B. bacteriovorus* is liable to be phagocytosed by alveolar macrophages. In this study, we used an uncomplicated carrier (i.e., PMs) to address the problems of *B. bacteriovorus* survival in the lung where pulmonary macrophages are fulfilled, effective release of *B. bacteriovorus* from the carrier, and more importantly, wide distribution in the lung after inhalation. These advantages make one-shot administration successful to achieve highly efficient therapy of antimicrobial-resistant Gram-negative bacterial lung infection which troubles human beings in the past and present time. Besides the high therapeutic efficacy, safety is another major advantage of BPMs. Both the cargo, i.e., *B. bacteriovorus* and the carrier matrix, PLGA, do not take any damage to our body. BPMs are a promising live biotherapeutic product (LBP) against lung infects caused by Gram-negative bacteria although many evidences are needed.

## CRediT authorship contribution statement

**Yan Liu:** Writing – original draft, Methodology, Data curation, Conceptualization. **Wanmei Wang:** Methodology, Investigation, Formal analysis. **Ruiteng Li:** Methodology, Investigation, Formal analysis. **Hui Zhang:** Methodology, Investigation, Formal analysis. **Wanting Guo:** Methodology, Investigation, Formal analysis. **Bochuan Yuan:** Validation, Supervision, Conceptualization. **Lina Du:** Validation, Supervision, Conceptualization. **Yiguang Jin:** Writing – review & editing, Supervision, Resources, Conceptualization.

## Declaration of competing interest

We declare that we have no known competing financial interests or personal relationships that could have appeared to influence the work reported in this paper.

## Data Availability

Data will be made available on request.
