# Supplementary material for: Inhaled predatory bacteria-loaded large porous microspheres to eradicate drug-resistant Pseudomonas aeruginosa from the lung
Source: Mater Today Bio. 2025 Feb 8;31:101562. doi: 10.1016/j.mtbio.2025.101562 (PMC11870198; doi:10.1016/j.mtbio.2025.101562)
Supplement: Multimedia component 1 [file mmc1.docx]

**Supplementary Material**

**Inhaled predatory bacteria-loaded large porous microspheres to eradicate drug-resistant *Pseudomonas aeruginosa* from the lung**

**Yan Liu^a,b^, Wanmei Wang^a^, Ruiteng Li^a^, Hui Zhang^a^, Wanting Guo^a^, Bochuan Yuan^a^, Lina Du^a^, Yiguang Jin^a,^***

^a^*Beijing Institute of Radiation Medicine, Beijing 100850, China*

^b^*School of Pharmacy, Xuzhou Medical University, Xuzhou 221004, China*

*Corresponding author.

E-mail address: jinyg@sina.com (Yiguang Jin).


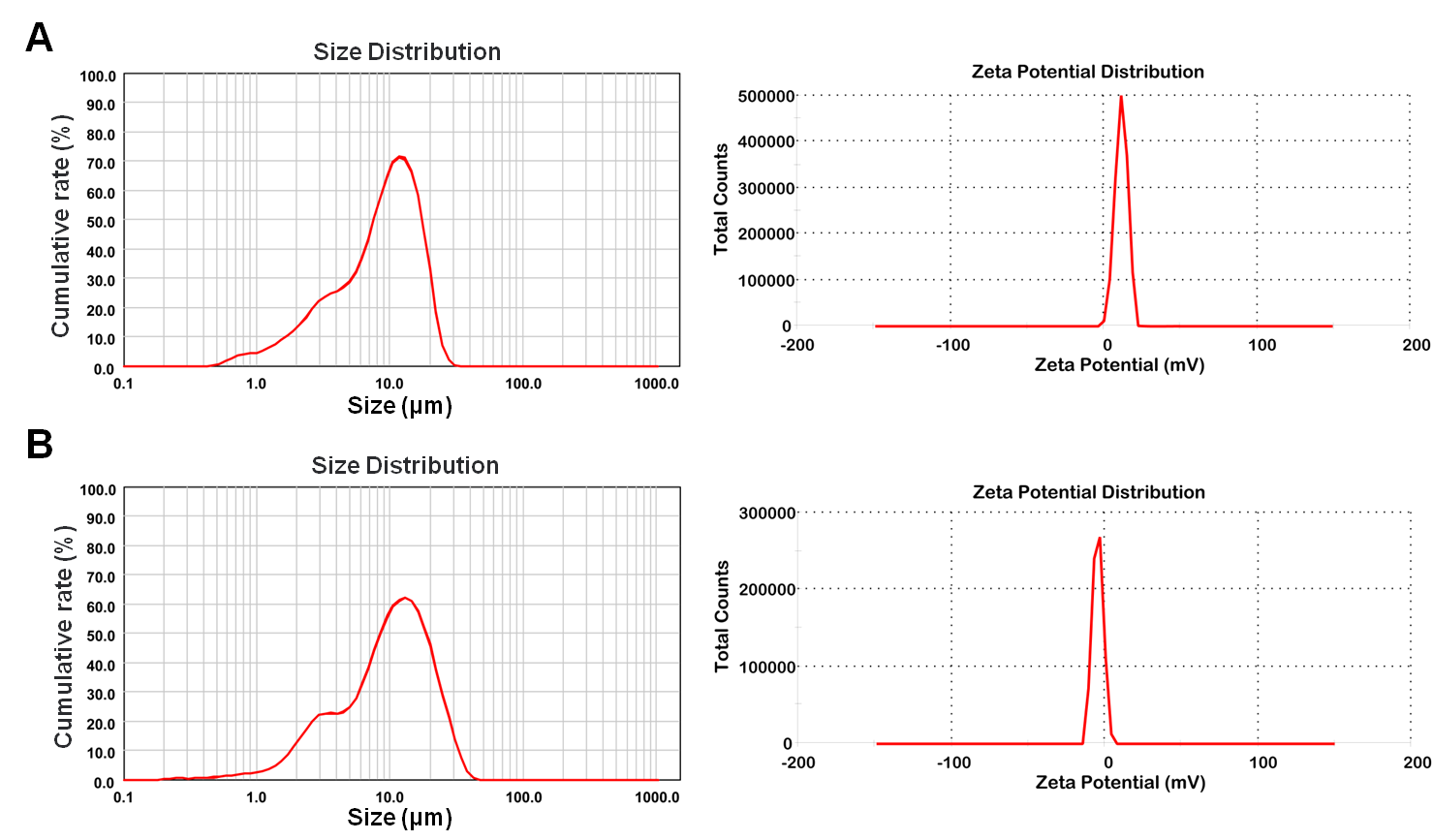


**Fig. S1.** Particle size and zeta potential of microspheres. (A) Particle size distribution and zeta potential of PMs. (B) Particle size distribution and zeta potential of BPMs.


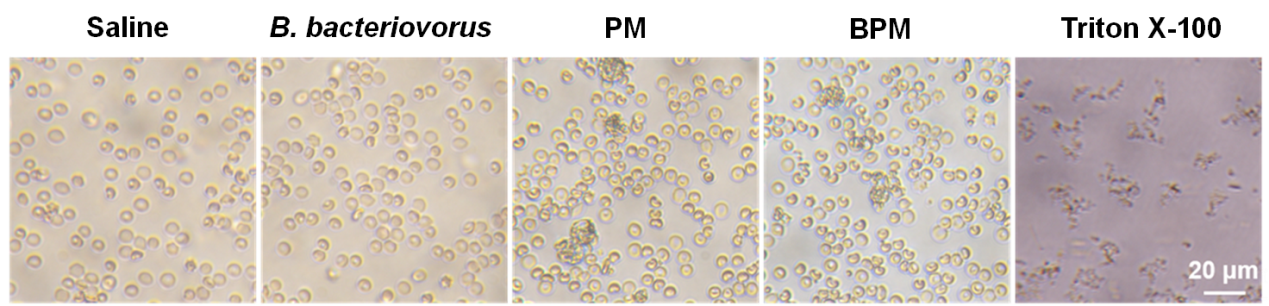


**Fig. S2.** Microscopic images of red blood cells after mixing with different components.


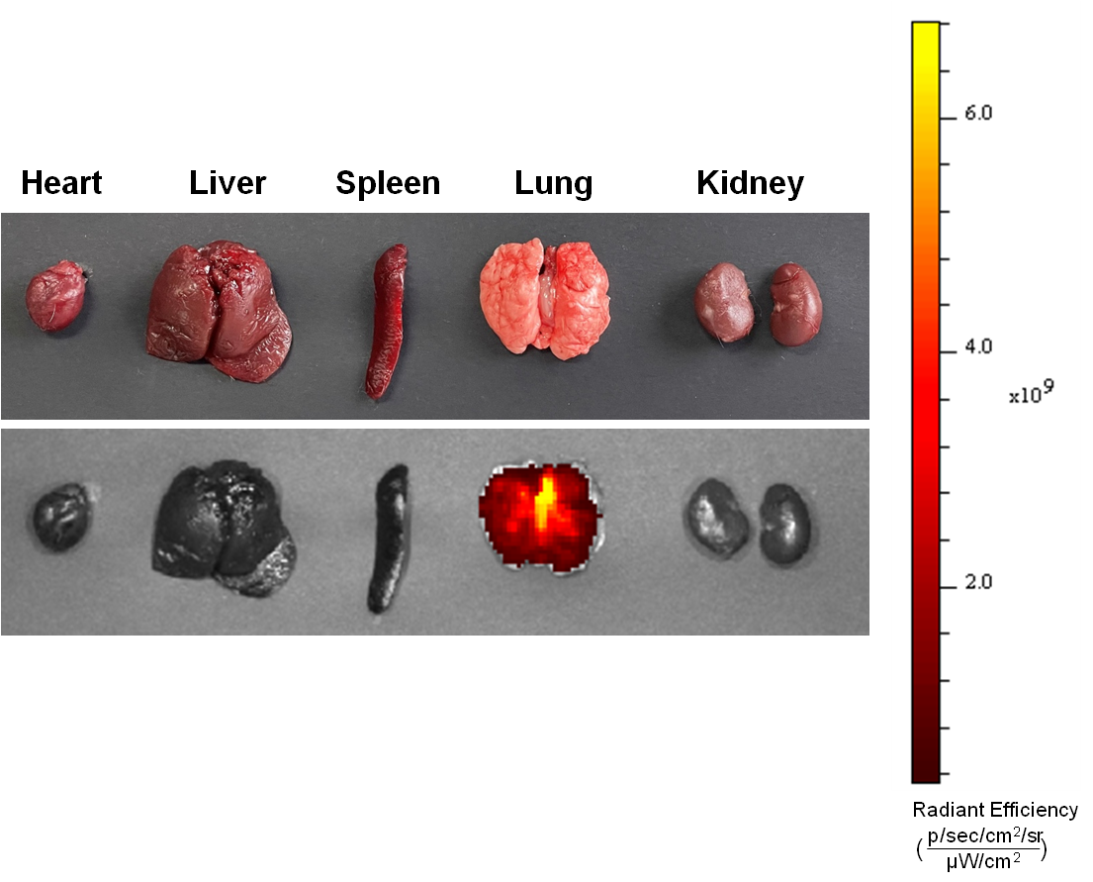


**Fig. S3.** *In vivo* distribution of Cy7-labeled BPMs. IVIS images of the distribution of Cy7-labeled BPMs in different organs after i.t. administration to rats for 4 h.


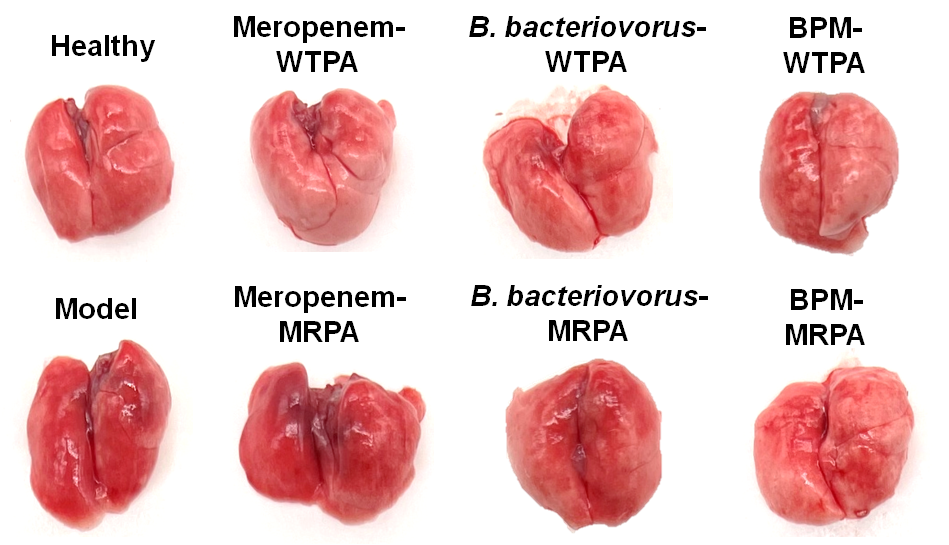


**Fig. S4.** Appearance of lung tissues in different groups of mice. Meropenem-WTPA, *B. bacteriovorus*-WTPA, and BPM-WTPA represent mice with WTPA pneumonia treated with meropenem solutions, *B. bacteriovorus* suspensions, and BPM suspensions, respectively. Meropenem-MRPA, *B. bacteriovorus*-MRPA, and BPM-MRPA represent mice with MRPA pneumonia treated with meropenem solutions, *B. bacteriovorus* suspensions, and BPM suspensions, respectively.
